# Intestinal bacteria flora changes in patients with *Mycoplasma pneumoniae* pneumonia with or without wheezing

**DOI:** 10.1038/s41598-022-09700-0

**Published:** 2022-04-05

**Authors:** Yonghong Jiang, Chunxiu Bao, Xiaoyang Zhao, Yiliu Chen, Yao Song, Zhen Xiao

**Affiliations:** Department of Paediatrics, Longhua Hospital Affiliated to Shanghai University of Traditional Chinese Medicine, No.725 South Wanping Road, Xuhui District, Shanghai, 200032 China

**Keywords:** Microbiology, Diseases, Medical research

## Abstract

*Mycoplasma pneumoniae* (MP) infection is a common cause of community-acquired pneumonia in children. Furthermore, many children with *Mycoplasma pneumoniae* pneumonia (MPP) have recurrent wheezing and reduced small airway function after their clinical symptoms have resolved, eventually leading to asthma. MPP can trigger immune disorders and systemic inflammatory responses. Hence, the intestine is the largest immune organ of the body. Therefore, we sought to investigate whether the alteration of intestinal flora is correlated with the development of wheezing in children with MPP. We collected 30 healthy children as group A, 50 children with nonwheezing MPP as group B, and 50 children with wheezing MPP as group C. We found that the percentage of eosinophil cells (EC) was significantly higher in group C than that in group B for routine blood tests and serum inflammatory factors. The serum cytokines, including IL-4, IL-17, TNF-α, and TGF-β, were significantly higher in group C than in group B. In addition, the level of IL-10 was significantly lower in group C than in group B. The distribution characteristics of intestinal flora strains in children with MPP were detected by sequencing of 16S rRNA gene amplicon sequencing. There were differences in the abundance of intestinal flora between children with MPP and healthy children, with lower abundance of *Ruminococcus flavefaciens*, *Clostridium butyricum*, *Lactobacillus*, and *Bifidobacterium* in the intestine of children with MPP compared to healthy children. The abundance of *Ruminococcus flavefaciens* and *Clostridium butyricum* was significantly lower in the intestine of children with wheezing MPP compared to children without wheezing MPP. In the correlation analysis between children with MPP and inflammatory factors, *Ruminococcus flavefaciens* was found to be negatively correlated with IL-17. *Clostridium butyricum* was negatively correlated with L-4, IL-17, TNF-α, and TGF-β; however, it positively correlated with IL-10. Thus, it was concluded that alterations in intestinal flora play a crucial role in the immune response to MPP, where a significant decline in intestinal *Ruminococcus flavefaciens* and *Clostridium butyricum* leads to an exacerbation of the inflammatory responses, which may promote the development of children with wheezing MPP.

## Introduction

*Mycoplasma pneumoniae* (MP) is a small, non-living microorganism that is intermediate between bacteria and viruses. MP infection accounts for 20%–40% of community-acquired pneumonia^1,2^. *Mycoplasma pneumoniae* pneumonia (MPP) can cause immune disorders and systemic inflammatory responses, leading to its tendency to persist and recur. This can progress to occlusive fine bronchitis and can trigger asthma^3^. Severe MPP can cause multisystem damage. In severe cases, respiratory and heart failure, and possibly death, may occur^4,5^. The pathogenesis of wheezing triggered by MP is complex, including autoimmune diseases, genetics, and environmental factors. Disorders of the factor gut flora are all associated with wheezing in patients with MP infection^6,7^.

The intestine is the largest immune organ of the body. The gut is the most microbially rich part of the body, with approximately 10^14^ bacteria present in the human gut and 400–1000 species that can be cultured with current technology^8–10^. The balance of such a large internal microflora can influence the occurrence, development, and prognosis of lung diseases^11^.

The intestinal microbial population plays a crucial role in the immune response to respiratory infections^12^. Respiratory infections cause alterations in the gut flora and changes in gut function. In addition, dysbiosis of the gut flora can exacerbate respiratory disease by altering the lung microbiota and even become a poor prognostic factor for respiratory disease^13^. Research on the gut microbiota has made great progress in the last decade, with many studies on gut flora and respiratory tract infections. However, most of the current studies have focused on viral and bacterial infections of the respiratory tract. There is a lack of reports of studies related to gut microbes and MPP, in which the molecular regulation of MPP pathogenesis mediated by gut microbes is a blind area. Therefore, we hypothesized that MP infection triggers immune disorders, immune disorders lead to gut flora dysbiosis, and flora dysbiosis in turn feedbacks to inflammatory amplification. Therefore, we started from the intestinal flora to study the characteristics of intestinal flora strains and the correlation of inflammatory factors in children with MPP and furthermore, to investigate whether the alteration of intestinal flora is correlated with the development of wheezing in children with MPP.

## Results

### Comparison of clinical and laboratory characteristics between children with MPP and healthy children

A total of 130 study samples were enrolled, including 30 healthy children (group A), 50 children with nonwheezing *Mycoplasma pneumoniae* pneumonia (MPP) (group B), and 50 children with wheezing MPP children (group C). The characteristics of the subjects are shown in Table 1. There were no statistically significant differences in the baseline characteristics between children with MPP and healthy children. Moreover, the three groups were comparable. Compared with the routine blood test of children with MPP, the percentage of eosinophil cell (EC) in group C was significantly higher than that in group B (*P* < *0.05*). Furthermore, there were no significant differences in other indices. The serum cytokine levels (IL-4, IL-17, TNF-α, and TGF-β) were significantly higher in group C than in group B (*P* < *0.001*). The serum IL-10 levels were also significantly lower in group C than in group B (*P* < *0.001*).

**Table 1:** Characteristics of subjects in the study.

Characteristics	A (*n* = 30)	B (*n* = 50)	C (*n* = 50)	*P*-value
Baseline characteristics of MPP children and healthy children (mean ± SD)				
Age	4.63 ± 1.13	4.68 ± 1.12	4.56 ± 1.16	0.869
BMI	15.93 ± 0.69	15.94 ± 0.86	15.91 ± 0.79	0.981
Gender				0.767
Male	16	23	26	
FEMALE	14	27	24	
Whole blood cell analysis (mean ± SD)				
White blood cell (× 10^9^ )		8.66 ± 3.92	24.77 ± 11.82	0.332
Neutrophils (%)		72.25 ± 93.56	58.32 ± 17.00	0.303
Lymphocytes (%)		39.60 ± 14.51	29.95 ± 16.12	0.833
Mononuclear cell (%)		7.91 ± 3.16	8.49 ± 3.07	0.355
Eosinophil cell (%)		1.68 ± 2.48	2.67 ± 2.43	0.043
Blood platelet (× 10^9^ )		247.44 ± 73.49	257.01 ± 79.44	0.531
C-reactive protein (mg/L)		8.81 ± 13.70	15.36 ± 37.51	0.253
Expression of mammary immune-associated factors [Median(inter-quartile range)]				
IL-4 (ng/L)		234.45 (90.71)	298.79 (103.12)	0.001
IL-10 (ng/L)		50.72 (23.20)	37.12 (18.81)	0.001
IL-17 (ng/L)		17.60 (7.10)	32.81 (9.64)	0.001
TNF-α (ng/L)		119.55 (54.70)	148.01 (52.23)	0.001
TGF-β (ng/L)		7899.17 (3031.21)	10,220.46 (3392.79)	0.001

BMI, body mass index; MPP, *Mycoplasma pneumoniae* pneumonia; Interleukin, IL; TNF-α, Tumor necrosis factor-α; TGF-β, transforming growth factor-β.

### Sample operational taxonomic unit (OTU) classification

Statistical analysis of operational taxonomic unit (OTU) was carried out based on a 97% similarity level. In the 130 stool samples, the distribution of clean tags after quality control was between 49,196, and 89,260. The number of valid tags (i.e., data used for final analysis), after chimeras were removed from clean tags, was between 45,689 and 86,157. The mean length of valid tags was 405.75–425.1 bp, and the number of OTU per sample was 422–3,152.

### Species classification and abundance analysis

**Figure 1:**
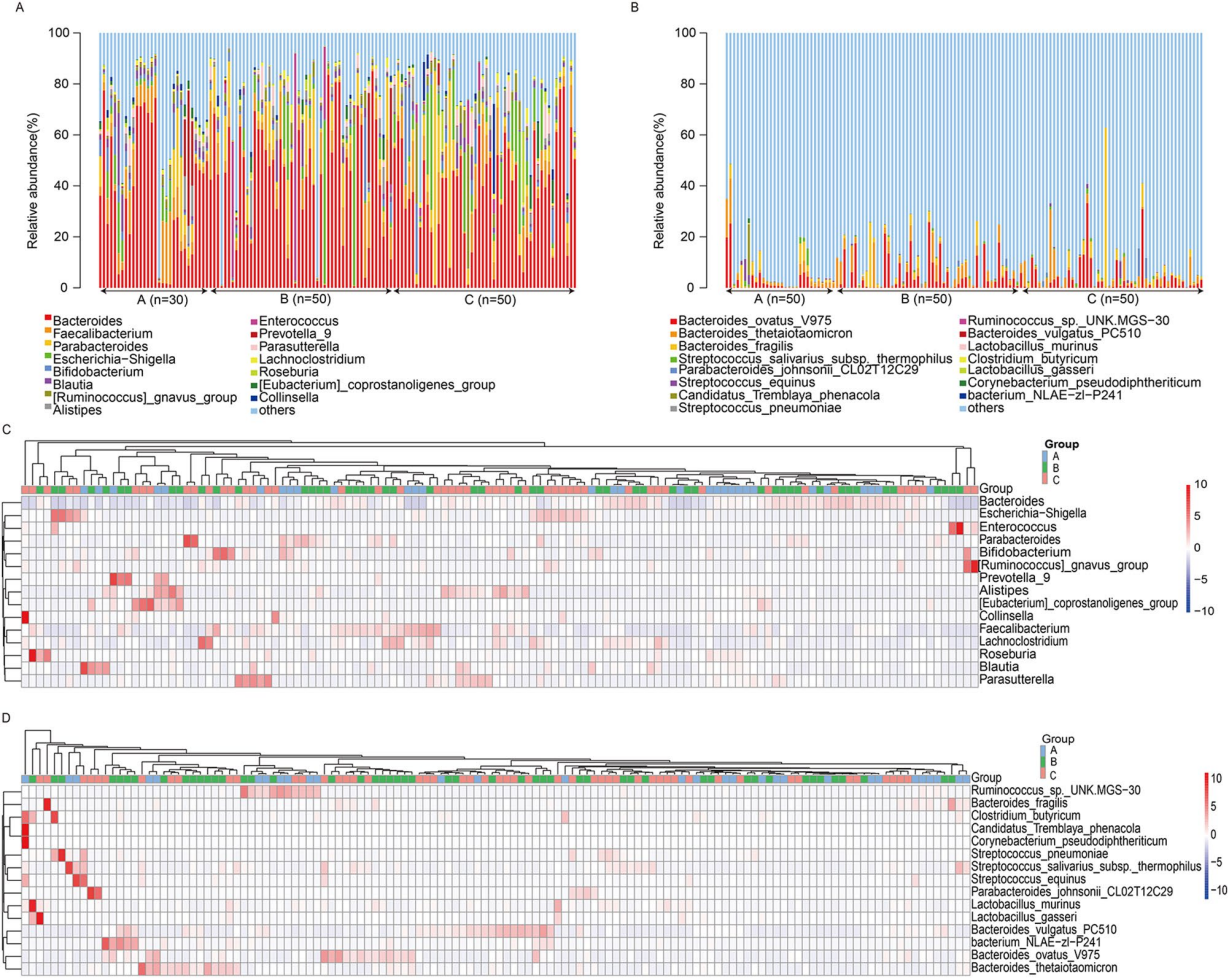
Microbiota profiles in healthy and MPP child. Top 15 abundant microbiota in healthy (**A**, *n* = 30), nonwheezing MPP (**B**, *n* = 50), and wheezing MPP (**C**, *n* = 50) child at the (a) genus and (b) species levels. Heatmap of top 15 abundant microbiota in healthy child (**A**, *n* = 30), child with nonwheezing MPP (**B**, *n* = 50), and child with wheezing MPP (**C**, *n* = 50) at the (c) genus and (d) species levels.

At the genus level, 1,056 genera were detected in samples from the three groups. Figure 1a shows the top 15 genera by abundance, of which genera with a relative abundance of > 3% include *Bacteroides* (38.78%), *Faecalibacterium* (7.07%), *Parabacteroides* (6.06%), *Escherichia-Shigella* (5.22%), and *Bifidobacterium* (3.17%).

In healthy children samples, 808 genera were common in all 30 samples, and genera with a relative abundance of > 3% included the following: *Bacteroides* (35.67%), *Faecalibacterium* (8.64%), *Parabacteroides* (4.42%), *Bifidobacterium* (3.98%), *Blautia* (3.70%), *Prevotella 9* (3.50%), and *Escherichia-Shigella* (3.26%). In children with nonwheezing MPP samples, 939 genera were common in 50 samples and genera with a relative abundance of > 3% included the following: *Bacteroides* (41.88%), *Faecalibacterium* (6.92%), *Parabacteroides* (6.09%), *Escherichia-Shigella* (4.72%), and *Enterococcus* (3.64%). In children with wheezing MPP samples, 898 genera were common in 50 samples and genera with a relative abundance of > 3% included the following: *Bacteroides* (37.54%), *Parabacteroides* (7.03%), *Escherichia-Shigella* (6.90%), *Faecalibacterium* (6.27%), *Bifidobacterium* (3.71%), and *Ruminococcus gnavus* (3.53%).

At the species level, 301 species were detected. Figure 1b shows the top 15 species by abundance, of which species with a relative abundance of > 3% included *Bacteroides ovatus V975* (4.22%).

In healthy children samples, 204 species were common in the 30 samples, and species with a relative abundance of > 3% included the following: ambiguous taxa (11.76%) and uncultured bacteria (10.96%). In children with nonwheezing MPP samples, 261 samples were common in the 50 samples and species with a relative abundance of > 3% included the following: ambiguous taxa (9.87%), uncultured bacteria (9.09%), *Bacteroides ovatus V975* (5.31%), and *Bacteroides thetaiotaomicron* (3.02%). In children with wheezing MPP samples, 233 species were common in the 50 samples and species with a relative abundance of > 3% included the following: ambiguous taxa (14.61%), uncultured *bacterium* (8.76%), and *Bacteroides ovatus V975* (3.96%).

The heatmap of the top 15 abundant microbiota among three groups, at the genus and species levels, was shown in Fig. 1c, d. At the species level, the relative abundance of *Ruminococcus_*sp.*_UNK.MGS-30* and *Clostridium butyricum* was decreased in children with MPP compared with healthy children, of which the decrease was more significant in children with wheezing MPP.

**Figure 2:**
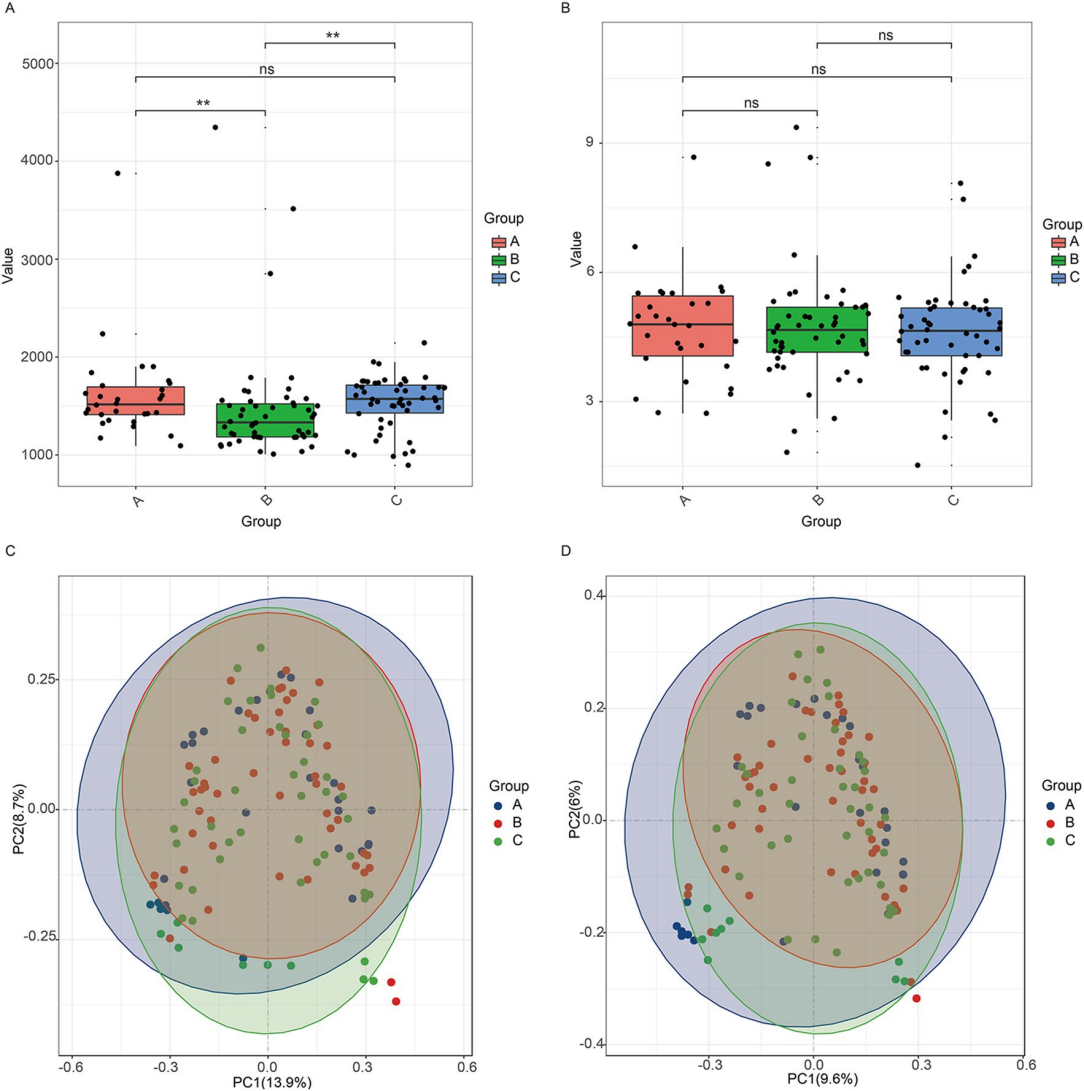
α- and β-diversity showing the overall comparison of the microbiota. (a) Chao index and (b) Shannon index in healthy child (**A**, *n* = 30), child with nonwheezing MPP (**B**, *n* = 50), and child with wheezing MPP (**C**, *n* = 50). PCoA plots based on (c) Bray–Curtis and (d) Binary-Jaccard dissimilarities showing the clustering of the bacterial community compositions for healthy child, child with nonwheezing MPP, and child with wheezing MPP. One-way ANOVA test was used for a and b. **P < 0.01. ns, not significant.

The species richness estimators by Chao and the species diversity indexes by Shannon were adapted to analyze the biodiversity in this study. The Chao index clearly showed that the community richness of samples from children with nonwheezing MPP was lower than that of the samples from healthy children (Fig 2a). The community richness of samples from children with wheezing MPP was higher than that of the samples from children with nonwheezing MPP children (Fig. 2a). However, the Shannon index revealed no significant difference in the community diversity among the samples from the three groups (Fig. 2b).

As shown on the principal coordinate analysis (PCoA) scatter plot based on Bray–Curtis (Fig. 2c) or Binary-Jaccard distance matrices (Fig. 2d), PC1 explained 13.9% and 9.6% of the variance observed, while PC2 explained 8.7% and 6% of the variance observed. Moreover, Adonis results, in which *p* = 0.002 for Bray–Curtis and *p* = 0.001 for Binary-Jaccard, confirmed the findings that the three groups were clearly divided.

### Multivariate statistical analysis of microorganisms

**Figure 3:**
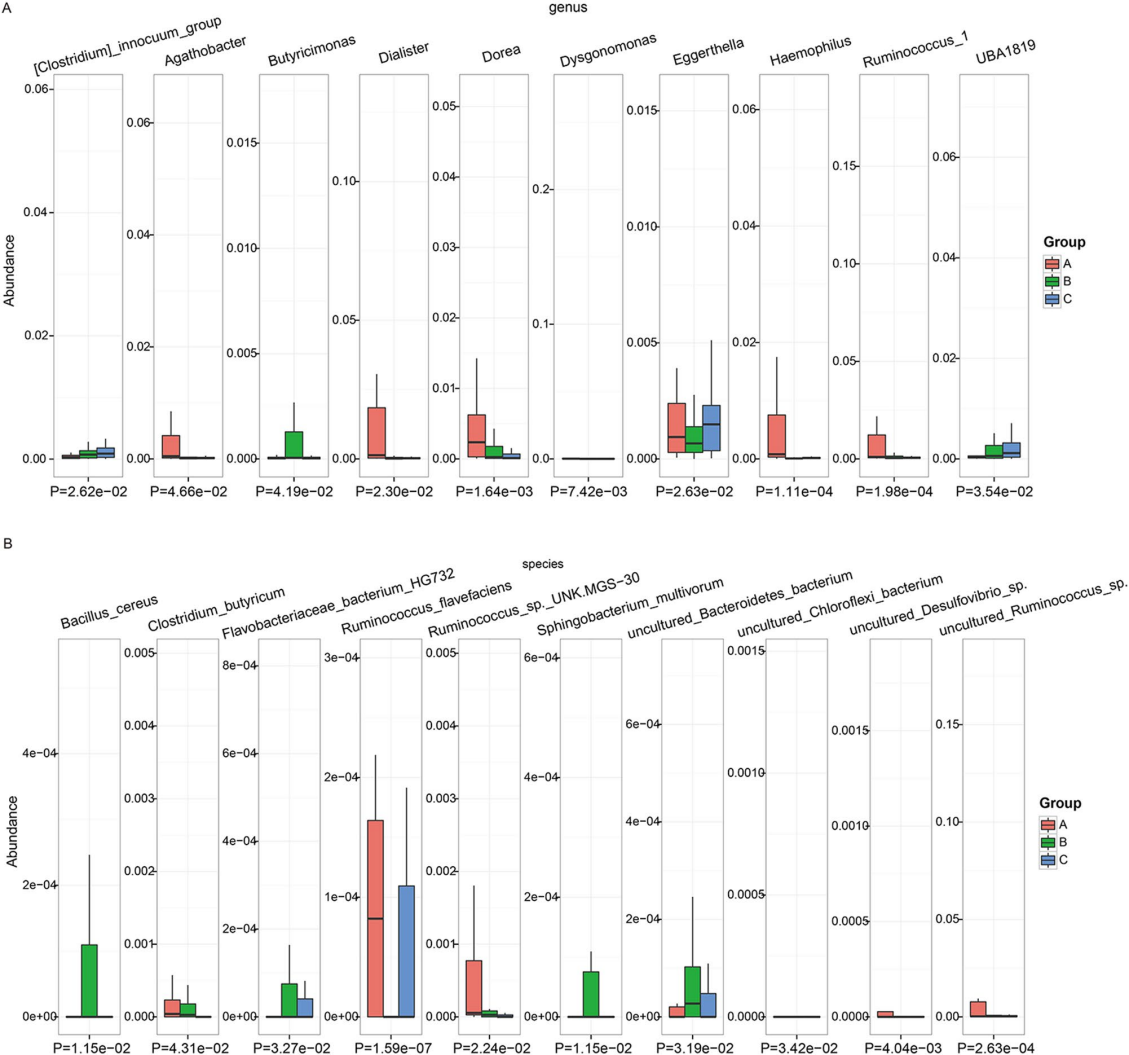
Top 10 abundant microbiota in healthy and MPP child. Top 10 abundant microbiota in healthy child (**A**, *n* = 30), child with nonwheezing MPP (**B**, *n* = 50), and child with wheezing MPP (**C**, *n* = 50) at the (a) genus and (b) species levels. One-way ANOVA test was for a and b.

Figure 3a shows the top 10 differential genera by abundance based on a one-way analysis of variance (ANOVA). The abundances of *Agathobacter*, *Dialister*, *Dorea*, *Eggerthella*, *Haemophilus*, *Ruminococcus 1*, and *Dysgonomonas* were decreased in children with nonwheezing MPP than in healthy children. In addition, the abundances of *Ruminococcus 1*, *Dorea*, *Dysgonomonas*, and *Butyricimonas* were decreased in children with wheezing MPP than in children with nonwheezing MPP.

Figure 3b shows the top 10 differential species by abundance based on a one-way ANOVA. Compared with healthy children, the abundances of *Ruminococcus flavefaciens*, uncultured *Ruminococcus* sp*.*, uncultured *Desulfovibrio* sp*.*, *Ruminococcus* sp*.* UNK.MGS-30, uncultured *Chloroflexi bacterium*, and *Clostridium butyricum* were decreased, whereas the abundances of *Bacillus cereus*, *Sphingobacterium multivorum*, uncultured *Bacteroidetes bacterium*, and *Flavobacteriaceae bacterium HG732* were increased in children with nonwheezing MPP. In addition, the abundances of uncultured *Ruminococcus* sp*.*, *Ruminococcus* sp*. UNK.MGS-30*, *Clostridium butyricum*, *Bacillus cereus*, *Sphingobacterium multivorum*, uncultured *Bacteroidetes bacterium*, and *Flavobacteriaceae bacterium HG732* were decreased in children with wheezing MPP than in children with nonwheezing MPP.

**Figure 4:**
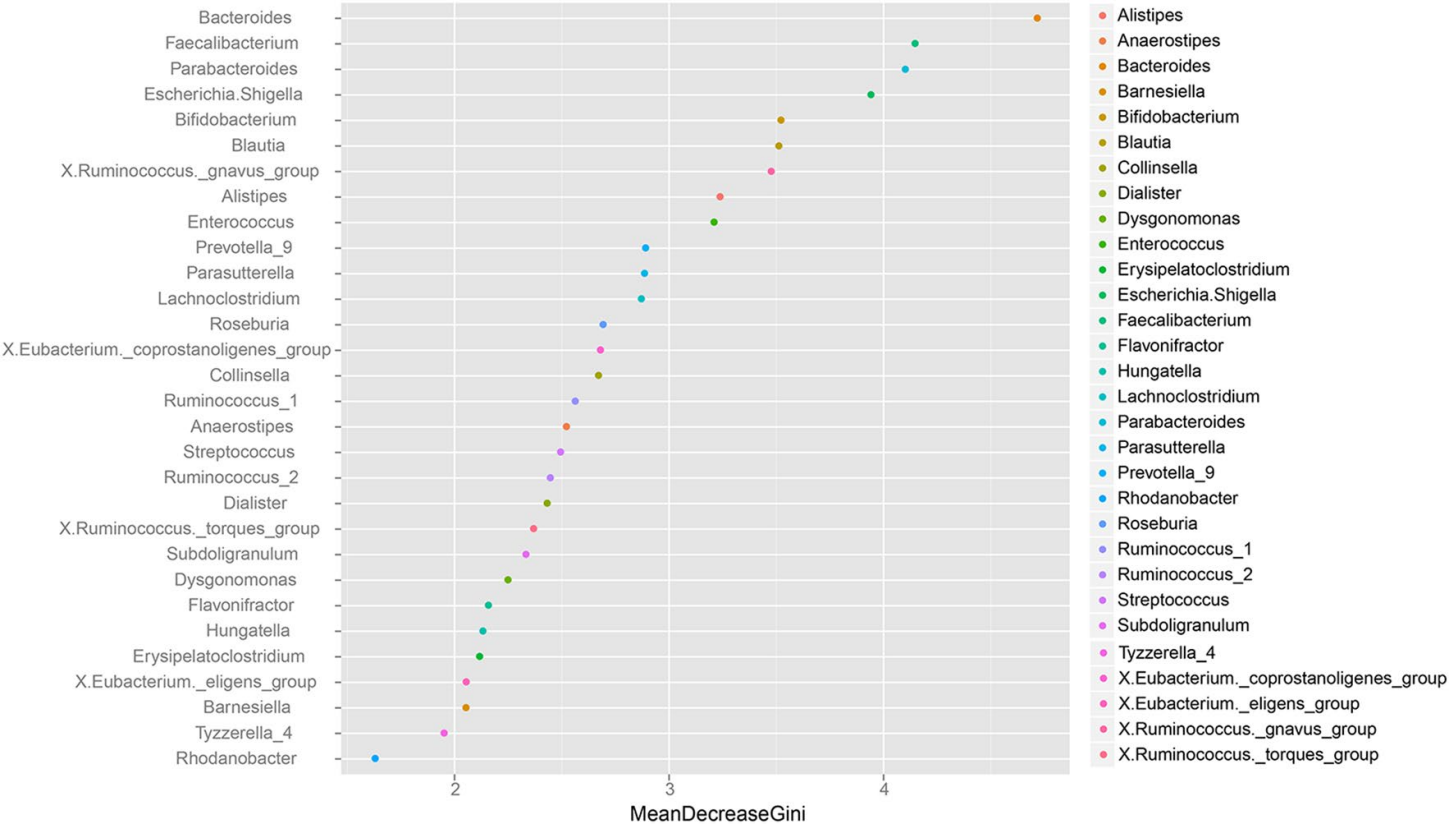
Two indexes, namely, the mean decrease in Gini, was calculated with a trained “random forest” model, which revealed the essential genera that triggered the difference among healthy child, child with nonwheezing MPP, and child with wheezing MPP.

Random forest classification is a widely used machine learning algorithm, which can be applied in the study of microbiology to identify important variables between experimental groups. In this study, the top 30 dominant genera in all the samples were selected to train the random forest model. The mean decrease in Gini value was reliable and a relevant predictor for performing classifications, which was used to identify the most essential genera among the three groups. Higher values in the decrease of Gini value represent the higher importance of a genus (Fig. 4).

### Phylogenetic investigation of communities by reconstruction of unobserved states (PICRUSt) analysis

**Figure 5:**
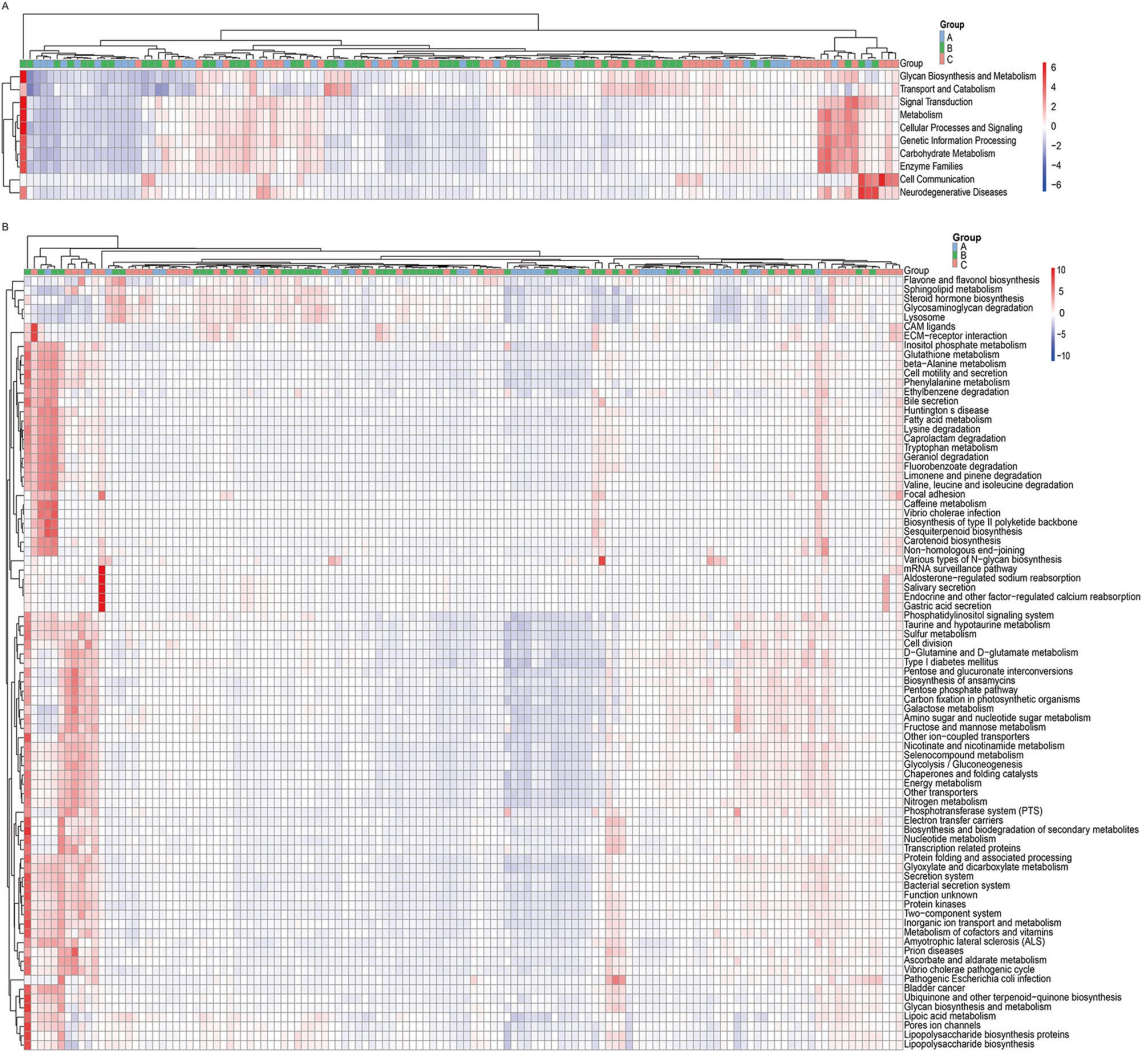
Heatmap of abundance of pathways in KEGG database. Abundance of different pathways based on KEGG (a) level 2 and (b) level 3 in healthy child (**A**, *n* = 30), child with nonwheezing MPP (**B**, *n* = 50), and child with wheezing MPP (**C**, *n* = 50).

There were ten metabolic pathways in KEGG level 2 abundance analysis, including glycan biosynthesis and metabolism, cell communication, neurodegenerative diseases, transport and catabolism, signal transduction, carbohydrate metabolism, metabolism, cellular processes and signaling, genetic information processing, and enzyme families (Fig 5a). There were 83 metabolic pathways in KEGG level 3 abundance analysis. The top 10 metabolic pathways including various types of N-glycan biosynthesis, Huntington’s disease, CAM ligands, ECM-receptor interaction, mRNA surveillance pathway, electron transfer carriers, biosynthesis, and biodegradation of secondary metabolites, focal adhesion, nucleotide metabolism, and caffeine metabolism were identified (Fig. 5b). Compared with healthy children, the abundances of four metabolic pathways were decreased in children with nonwheezing MPP, including various types of N-glycan biosynthesis, caffeine metabolism, biosynthesis of type II polyketide backbone, and sesquiterpenoid biosynthesis. Compared with children with nonwheezing MPP, the abundances of 27 metabolic pathways decreased in children with wheezing MPP, of which the top 10 metabolic pathways included caffeine metabolism, *Vibrio cholerae* infection, biosynthesis of type II polyketide backbone, sesquiterpenoid biosynthesis, CAM ligands, ECM-receptor interaction, lipopolysaccharide biosynthesis proteins, steroid hormone biosynthesis, ubiquinone and other terpenoid-quinone biosynthesis, and bile secretion.

**Figure 6:**
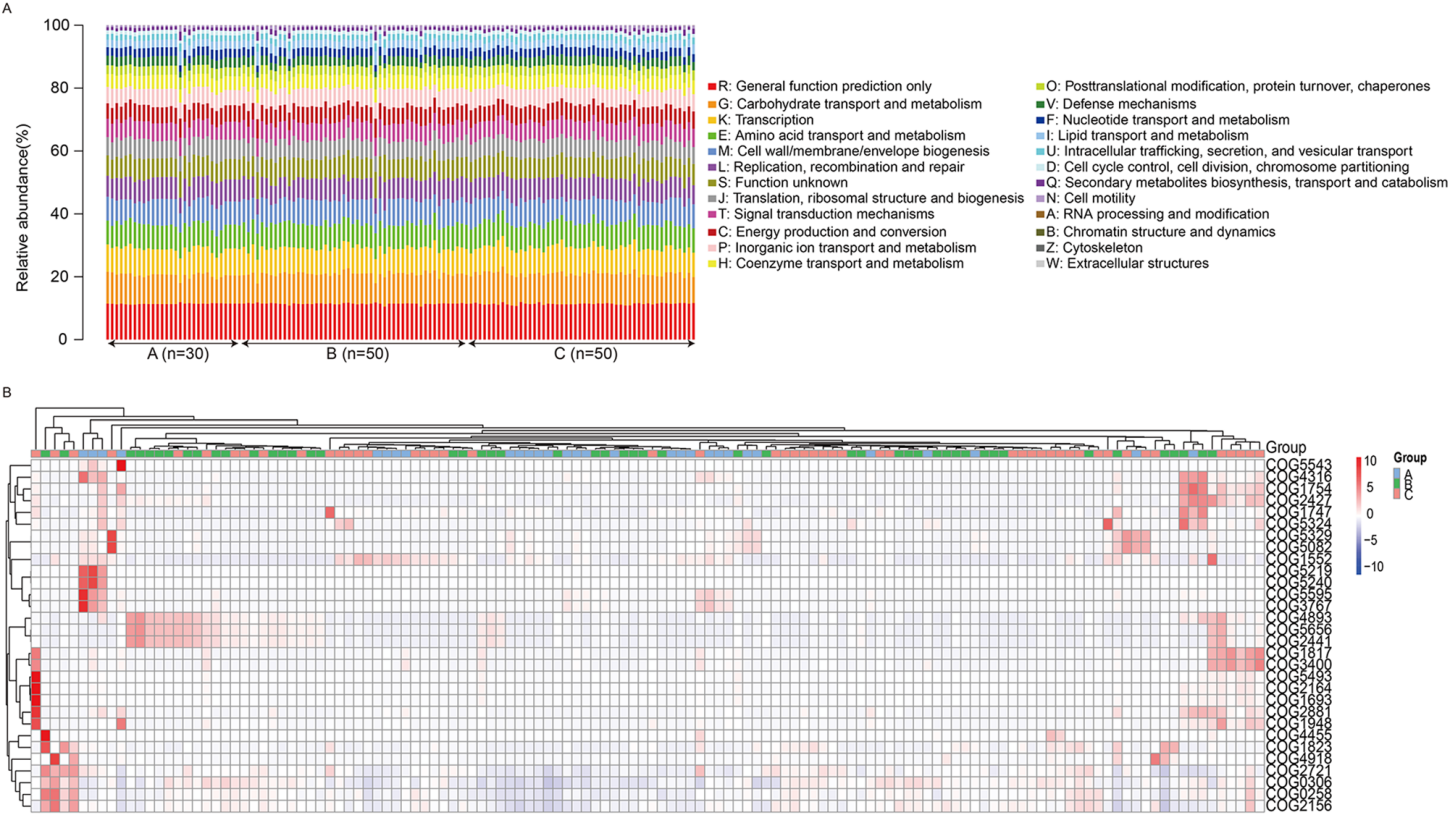
COG functional classification in healthy and MPP patients. Relative abundance of different COG functional classification in healthy child (**A**, *n* = 30), child with nonwheezing MPP (**B**, *n* = 50), and child with wheezing MPP (**C**, *n* = 50). Heatmap of top 30 abundant COG functional classification in healthy child (**A**, *n* = 30), child with nonwheezing MPP (**B**, *n* = 50), and child with wheezing MPP (**C**, *n* = 50).

The Cluster of Orthologous Group (COG) function prediction showed that the COG functional composition was highly similar in samples from the three groups, and the functional annotation results could be divided into 24 categories. The top five categories of high functionality were general function prediction only, carbohydrate transport and metabolism, transcription, amino acid transport and metabolism, and cell wall/membrane/envelope biogenesis (Fig. 6a). In addition, 4,112,853 genes with unknown function were found, which will be studied in the future. Compared with healthy children, the abundances of six COG categories were decreased in children with nonwheezing MPP children, including replication, recombination, and repair; translation, ribosomal structure, and biogenesis; defense mechanisms; cell cycle control, cell division, chromosome partitioning; chromatin structure and dynamics; and extracellular structures. Compared with children with nonwheezing MPP, the abundance of the cytoskeleton was decreased in children with wheezing MPP. The heatmap of the top 30 abundant COG functional classification in the three groups was shown in Fig. 6b.

### Real-time quantitative polymerase chain reaction (qRT-PCR) validation of DNA copy numbers of Ruminococcus flavefaciens, Clostridium butyricum, Lactobacillus, and Bifidobacterium

**Figure 7:**
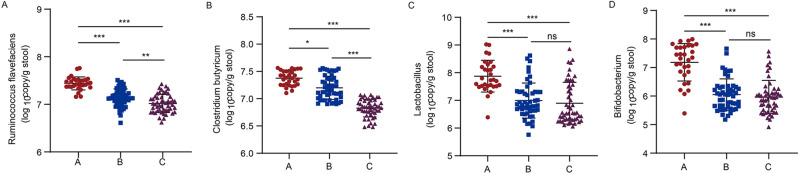
DNA copy numbers of *Ruminococcus flavefaciens*, *Clostridium butyricum*, *Lactobacillus*, and *Bifidobacterium*. DNA copy numbers of (a) *Ruminococcus flavefaciens*, (b) *Clostridium butyricum*, (c) *Lactobacillus*, and (d) *Bifidobacterium* in healthy child (**A**, *n* = 30), child with nonwheezing MPP (**B**, *n* = 50), and child with wheezing MPP (**C**, *n* = 50) were measured by qRT-PCR. One-way ANOVA test was used for a and Kruskal–Wallis test was used for b–d. *P < 0.05, **P < 0.01, ***P < 0.001. ns, not significant.

Among the three groups, the levels of four species were decreased in children with MPP than in healthy children. Thus, the decrease in *Ruminococcus flavefaciens* and *Clostridium butyricum* was more significant in children with wheezing MPP (Figure. 7a–d), but the differences in the DNA copy numbers of *Lactobacillus* and *Bifidobacterium* between children with wheezing MPP and children with nonwheezing MPP were not significant.

### Correlation analysis between gut microflora and serum inflammatory factors in children with MPP

The correlation between intestinal bacteria flora (*Bifidobacterium*, *Lactobacillus*, *Ruminococcus flavefaciens*, and *Clostridium butyricum*) and serum inflammatory factors (IL-4, IL-10, IL-17, TNF-α, and TGF-β) in 100 children with MPP was shown in Table 2. *Ruminococcus flavefaciens* was negatively correlated with IL-17 content, with the correlation coefficient (*r*) = −0.286, *P* < *0.01*; *Clostridium butyricum* was negatively correlated with IL-4 (*r* = −0.361, *P* < 0.001), IL-17 (*r* = −0.544, *P* < 0.001), TNF-α (*r* = −0.328, *P* < 0.01), and TGF-β (*r* = −0.336, *P* < 0.01); *Clostridium butyricum* was positively associated with IL-10 content (*r* = 0.220, *P* < *0.05*), and there was no significant correlation between the other indices (*P* > *0.05*).

**Table 2:** Correlation coefficient of the number of intestinal bacteria flora and the serum inflammatory factor content.

Intestinal bacteria flora	Correlation coefficent (*r*)				
	IL-4	IL-10	IL-17	TNF-α	TGF-β
*Bifidobacterium*	–0.100	0.002	0.061	–0.074	0.075
*Lactobacillus*	–0.098	0.061	–0.007	0.084	0.032
*Ruminococcus flavefaciens*	–0.075	0.021	–0.286	–0.160	-0.097
*Clostridium butyricum*	–0.361	0.220	–0.544	–0.328	–0.336

## Discussion

MP is an extracellular pathogen lacking cell wall and is one of the common pathogens of respiratory tract infections in pediatrics. Pneumonia caused by MP accounts for 10%–40% of community-acquired pneumonia^1^. Recent research shows that MPP is not limited to school-age children and the prevalence in infants and young children is also rising^14^. The pathogenesis of MPP remains unclear. Some studies have shown that certain cytokines are activated, and associated inflammatory factors are secreted after children are infected with MP. Thus, this leads to a decrease in the immune function of the affected children^15^. Furthermore, the degree of their immune disorder is related to the severity of the disease in children with MPP^16^. The results of our study showed that EC% was significantly higher in children with wheezing MPP than in the non-snuffing group. The serum inflammatory factors, including IL-4, IL-17, TNF-α, and TGF-β levels, were significantly higher. Moreover, IL-10 levels were significantly lower. In our previous study^17^, similar results were obtained regarding the MPP inflammatory pathway. We found that the expressions of pro-inflammatory cytokines such as IL-6, IL-1 β, IL-17, TNF-α, and TGF-β were significantly increased after an MP infection. This indicates that many cellular inflammatory factors in the body of children with MPP are in a state of disorder and the immune disorder is even more severe in children with wheezing MPP who are in a state of hypersensitivity. When MP enters the body, it activates the body to produce pro- and anti-inflammatory cytokines, and the interaction between the two can affect the occurrence and development of the inflammatory response in the body. The body can produce pro-inflammatory cytokines such as IL-4, IL-17, TNF-α, and TGF-β to promote tissue repair by clearing pathogens; the body can also produce anti-inflammatory cytokines such as IL-10 to downregulate the body’s immune response and inhibit the overexpression of pro-inflammatory cytokines, thereby controlling the body’s inflammatory response and reducing the damage caused by pathogens^18^.

MPP can cause a systemic inflammatory response in the body, leading to immune disorders, which can trigger acute bronchial asthma attacks, exacerbate bronchial hyperresponsiveness, and even lead to severe or refractory asthma^19^. A study on wheezing and MP infection in infants showed that MP infection was closely associated with wheezing in infants^20^. Yehe et al.^21^ showed that children with MP-infected asthmatic wheeze were at risk of progression to asthma. Non-MP-infected pediatric patients and patients with atopic diseases (atopic exacerbations and allergic conjunctivitis) were at higher risk of MP-infection-induced wheeze. The exact role and pathogenesis of MP in asthma remain unclear. One study found that MP infection may lead to an imbalance in the Th1/Th2 cell ratio, resulting in Th2 cytokine dominance. This can stimulate the release of cytokines and inflammatory mediators such as TGF-β1, vascular endothelial growth factor, endothelin-1 and TNF-α, and eosinophil cationic proteins involved in asthma and airway remodeling, thereby inducing wheezing or exacerbating asthma^22^.

The intestinal tract is the largest immune organ of the body. Changes in the composition and function of the intestinal flora affect the respiratory tract through the mucosal immune system; on the other hand, disturbances in the respiratory flora also affect the digestive tract through an immune regulation. In the gut, more than 400 species of bacteria can be cultured with current technology, and the balance of these large internal microflorae can influence the onset, development, and prognosis of lung disease^11^. Several studies^23,24^ have demonstrated that pulmonary infections caused by bacteria and viruses can alter the microecological environment within the lower respiratory tract. This leads to changes and imbalances in the host gut microflora, accompanied by impaired mucosal barriers and immune function, further promoting the development of pulmonary infections. Studies on the role of gut microbes in pulmonary infections have been dominated by bacterial and viral infections; however, studies on the association of human MP infections with gut microbes have not been reported. In this study, the abundance and diversity of gut microorganisms in children with MPP significantly differed from those in healthy children, as determined by 16S rRNA sequencing. The top 10 groups with the most significant differences were *Ruminococcus flavefaciens*, *uncultured Ruminococcus* sp., *uncultured Desulfovibrio* sp., *Bacillus cereus*, *Sphingobacterium multivorum*, *Ruminococcus* sp. UNK.MGS-30, *uncultured Bacteroidetes bacterium*, *Flavobacteriaceae bacterium HG732*, and *uncultured Chloroflexi*. This suggests that MP infection can lead to an imbalance in the gut microbial balance in children with MPP, which is consistent with what is known about the effects of bacteria and viruses on the gut flora. Similarly, gut microbial populations can play a reverse regulatory role in the immune response to respiratory infections. The latest pneumonia treatment protocol for novel coronavirus infections (trial version 4 in January 27, 2020) released by the Chinese Health Care Commission specifically emphasizes the use of gut microecological regulators to maintain gut microecological homeostasis^25^. It suggested that after a new coronavirus infection, there is also often a microecological imbalance that leads to bacterial secondary infections, and the microecological balance can help reduce secondary infections^26^. A study showing the characteristics of the gut flora in patients with high SARS-CoV-2 virus infection suggested an increase in pathogenic bacteria, a decrease in beneficial bacteria, and a decrease in the temporal transcriptional activity of SARS-CoV2 and its association with alterations in the longitudinal fecal microbiota in patients with neocolonial pneumonia^27^. This suggests that there is an inextricable link between intestinal flora and lung infection. This link may be accomplished through immune regulation of the “gut-lung” axis.

In a study of gut flora in relation to allergy, it concluded that measures affecting microbial colonization in the first month of life might influence the development of asthma in children^28^. Transient changes in gut microbiota during the first 100 days of life were associated with a decrease in the number of bacteria belonging to the genera *Lachnospira*, *Veillonella*, *Faecalibacterium*, and *Rothia*, which can increase the risk of asthma^12^. Therefore, we hypothesized that changes in the intestinal flora may be associated with wheezing in children with MPP. The results of this study revealed the differences in the abundance of intestinal flora between children with MPP and healthy children, with lower intestinal abundance of *Ruminococcus flavefaciens*, *Clostridium butyricum*, *Lactobacillus*, and *Bifidobacterium* in children with MPP compared to healthy children. Children with wheezing MPP had significantly lower numbers of *Ruminococcus flavefaciens* and *Clostridium butyricum* compared to the non-wheezing group.

PICRUSt, based on annotated 16S sequencing data in the Greengenes database, was used to predict the composition of known microbial gene functions and to calculate the differences in functions between different samples and groups. Kyoto Encyclopedia of Genes and Genomes (KEGG) function prediction showed that the abundances of metabolic pathways, such as various types of N-glycan biosynthesis, caffeine metabolism, biosynthesis of type II polyketide backbone, and sesquiterpenoid biosynthesis, were decreased in children with nonwheezing MPP than in healthy children. Compared with nonwheezing MPP children, the abundances of metabolic pathways in the guts of children with wheezing MPP, such as caffeine metabolism, *Vibrio cholerae* infection, biosynthesis of type II polyketide backbones, sesquiterpenoid biosynthesis, CAM ligands, ECM-receptor interactions, lipopolysaccharide biosynthesis proteins, steroid hormone biosynthesis, ubiquinone and other terpenoid-quinone biosynthesis, and bile secretion, were decreased. COG functional prediction found that the abundances of replication, recombination, and repair; translation, ribosomal structure, and biogenesis; defense mechanisms; cell biogenesis; defense mechanisms; cell cycle control, cell division, and chromosome partitioning; chromatin structure and dynamics; and extracellular structures were decreased in children with nonwheezing MPP than in healthy children. The abundance of cytoskeleton was decreased in children with wheezing MPP than in children with nonwheezing MPP.

According to reports in recent years, the most commonly studied probiotics are *Lactobacillus* and *Bifidobacterium*. Their protective effects in animal models of respiratory virus infection have been well established^29^. For example, orally administered *Lactobacillus*, including *Lactobacillus casei*, *Lactobacillus rhamnosus*, *Lactobacillus gasseri*, *Lactobacillus pentosus*, *Lactobacillus plantarum*, *Lactobacillus brevis*, *Lactobacillus johnsonii*, *Bifidobacterium breve 155*, and *Bifidobacterium longum 180*, protected all the mice from influenza-virus-induced pathology and mortality. The main mechanisms include increased antibody production, enhanced natural killer cell activity, and increased IFN-γ and IL-10 by the probiotics^30^. This is consistent with the results obtained from our experiments. This study showed that the gut microorganisms of children with MPP had decreased DNA copy numbers of *Lactobacillus* and *Bifidobacterium* compared to healthy children. However, their differences in the gut of children with non-asthmatic and asthmatic MPP were not significant, so the effects of *Lactobacillus and Bifidobacterium* on children with asthmatic MPP need to be further investigated.

It has been pointed out that *Clostridium butyricum* has an extremely strong role in adjusting the balance of intestinal colonization; it can inhibit pathogenic bacteria in the intestine and promote the growth of beneficial bacteria such as *Bifidobacteria* and *Lactobacilli* in the intestine^31^. Sun et al.^32^ demonstrated that the amount of *Bifidobacteria* and *Lactobacilli* in the stool of patients with irritable bowel syndrome was reduced. After the treatment with *Clostridium butyricum*, there were no significant differences in *Bifidobacteria*, non-bacteriophage anaerobes, coliforms, *Enterococci*, and *Lactobacilli* between the stool and healthy individuals. Furthermore, clinical symptoms were significantly improved. Beyond the positive effects on intestinal tract, *Clostridium butyricum* addition was recently reported to regulate lipid metabolism in various tissues of broiler chickens^33^. In addition, *Clostridium butyricum* has been shown to promote intestinal motility by a regulation of TLR2 in interstitial cells of Cajal^34^. In this experiment, COG functional prediction revealed that the abundance of lipid transport and metabolism and the cell motility metabolic pathway was increased in the intestine of children with MPP compared to healthy children. Furthermore, the increase in the abundance of these two metabolic pathways was more pronounced in the intestine of children with wheezing MPP. Argonaute protein from the mesophilic bacterium *Clostridium butyricum* can utilize signal interfering DNA (siDNA) guides to cleave both single-stranded DNA (ssDNA) and double-stranded DNA (dsDNA) targets at moderate temperatures, mediating host defense against invading nucleic acids^35^. This is consistent with the results obtained from our experiments. The abundance of defense mechanisms in the gut of children with MPP was significantly reduced compared to healthy children, consistent with the reduced abundance of *Clostridium butyricum*.

*Ruminococcaceae* is together with *Lachnospiraceae*, the most abundant *Firmicute* families in the adult mammalian gut^36^, and has been associated with the maintenance of gut health. There is an emerging interest in the role of *Ruminococcus* colonization in infancy^37^. A possible health benefit is the production of *Ruminococcus*, such as ruminococcin A, a bacteriocin that can inhibit *Clostridium* species development^38^. A study of gut microbes in pigs following an MP infection suggests that the abundance and diversity of the gut microbiota may be a potential determinant of susceptibility to MP in pigs^39^. The abundance of *Ruminococcus gnavus* was associated with allergic diseases in infants^40^. These results are consistent with the results of our experiment. Moreover, 16S rRNA data showed that *Ruminococcus* counts were significantly decreased in the guts of pediatric patients with MPP compared with healthy children, particularly in pediatric patients with wheezing, which were consistent with the findings detected by qRT-PCR. *Ruminococcus* is also known to synthesize short-chain fatty acids, which may be one of the mechanisms for preventing MP or asthma. COG functional prediction showed that the abundance of fatty acid metabolism pathways was increased in the guts of children with MPP compared with healthy children. The increase in abundance was more significant in pediatric patients with wheezing MPP, which was inconsistent with the abundance of *Ruminococcus flavefaciens*, suggesting that other flora besides *Ruminococcus flavefaciens* may influence the fatty acid metabolism pathway. Therefore, a more in-depth mechanism research should be carried out in the future.

The intestinal flora of infants and children can have an impact on future lung health^41^. Bacterial flora penetrates through the intestinal and respiratory epithelium to colonize the infant’s body, thereby creating resistance to pathogens. Thus, dysbiosis of the intestinal flora can lead to altered immune responses in infants and children, which in turn can affect the development of lung diseases such as pulmonary cystic fibrosis degeneration through the “gut-lung axis.” In one report, a low relative abundance of *Ruminococcaceae* at 1 week of age in infants developed IgE-associated eczema. Furthermore, a low abundance of the genus *Ruminococcus* at that age was associated with exaggerated TLR-2-induced IL-6 and TNF-α response at 6 months^42^. It was noted that in the new-onset patients with pulmonary tuberculosis, thick-walled bacilli were negatively correlated with CD4^+^/CD8^+^ and CD14^+^CD16^+^ and are positively correlated with CD14^+^CD16^-^; at the genus level, *Enterococcus faecalis* was positively correlated with CD4^+^/CD8^+^ and CD4^+^^43^. Stimulation of predisposing immune responses against infection by MP, which involves induction of Th2 cytokines, immune cells, and IgE production, plays a role in causation of wheezing in patients with MPP^44^. As a polypeptide regulator of the immune response, TNF-α, which is produced by Th1 cells, can promote cell-mediated killing of intracellular pathogens^45^. IL-17 secreted by Th17 cells is involved in the cellular immune response and pathogen elimination, while the IL-4, TGF-β, and IL-10 cytokines secreted by Th2 and Treg cells have the effect of inhibiting the inflammatory response, and can antagonize the differentiation, maturation, and secretory function of Th1 and Th17^46^. In the present study, the serum cytokine levels (IL-4, IL-17, TNF-α, and TGF-β) were significantly higher in children with wheezing MPP, while the serum IL-10 levels were significantly lower in children with wheezing. This suggests that wheezing may be associated with the imbalance of Th1/Th2 and Th17/Treg cells in children with MPP. In the correlation analysis of intestinal flora and inflammatory factors in this study, *Ruminococcus flavefaciens* was found to be negatively correlated with IL-17. *Clostridium butyricum* was negatively correlated with IL-4, IL-17, TNF-α, and TGF-β, while it positively correlated with IL-10. In line with our findings, previous studies showed that *Ruminococcus flavefaciens* negatively correlated with proinflammatory cytokines^47^ and that *Clostridium butyricum* can suppress inflammatory responses by regulating the release of IL-4, IL-17, TNF-α, TGF-β and IL-10^48–50^. Taken together, MP infection triggers immune disorders, followed by dysbiosis of the intestinal flora, which in turn amplifies inflammatory response.

## Conclusions

We believe that our work has a role to play in the prevention and treatment of MP infections and in the maturation of the microbial “gut-lung axis,” which differs in the abundance and diversity of intestinal microorganisms in children with MPP compared to healthy children. We found a correlation between serum inflammatory factors and *Ruminococcus flavefaciens* and *Clostridium butyricum* in children with MPP and a significant decrease in the number of *Ruminococcus flavefaciens* and *Clostridium butyricum* in the gut of children with wheezing, which may be aggravates the inflammatory reaction of children with wheezing MPP. We suggest that the diversity of intestinal probiotics and the number of flora determine the microbial ecological balance of the gut in children and are relevant to the evolution and prognosis of children with MPP. We speculate that the “gut-lung axis” interrelation may also be related to gut flora metabolites, in addition to gut flora. In future studies of the gut flora of MPP, we will further develop the mechanisms of immune and metabolic interactions between the microflora in the gut and MP colonization in the lung. Studying the gut microecology in children, regulating the gut flora, and maintaining a balanced gut microecological environment may be a future trend in preventing asthma, and even respiratory disease in general, in children with MPP.

## Methods

### Approvals

This study was conducted at the Pediatrics Department of Longhua Hospital affiliated to the Shanghai University of Traditional Chinese Medicine. This study was approved by the ethics committee of Longhua Hospital affiliated to the Shanghai University of Traditional Chinese Medicine, and the ethics approval number is 2019LCSY018. All participating staff underwent relevant training before the trial including the peripheral blood samples, stool collection method, and inclusion and exclusion criteria to ensure data accuracy. The parents of all pediatric patients signed the informed consent forms, and all study tasks were carried out in accordance with the *Declaration of Helsinki* (1964).

### Patients

From September 2018 to January 2020, 100 hospitalized children with a definitive diagnosis of *Mycoplasma pneumoniae* pneumonia (MPP) in Longhua Hospital affiliated to the Shanghai University of Traditional Chinese Medicine were enrolled in the study, of which 50 had nonwheezing MPP and 50 had wheezing MPP. Thirty healthy children who met the criteria were enrolled in the study from Fenglin Road and Xietu Road communities in Xuhui District, Shanghai, China.

### Diagnostic criteria

The diagnostic criteria for mycoplasmal pneumonia in the 7th edition of Zhu Futang Practice of Pediatrics, edited by Yamei Hu and Zaifang Jiang, were used as the reference^51^ as follows: (1) persistent severe cough; (2) few lung signs, early and apparent chest X-ray changes; (3) penicillin, streptomycin, and sulfonamides ineffective for treatment; (4) normal or slightly elevated white blood cell count; (5) antibody positive for MP immunoglobulin M in the serum or serum cold agglutinin titer > 1:32 or mycoplasma-positive pharyngeal swabs, which can be used as a basis for definitive diagnosis.

### Inclusion and exclusion criteria

#### Inclusion criteria

Human gut microbiota composition and function are greatly affected by genetics, nutrition, environment, and geographical factors, particularly age, diet, obesity, and drugs^52^. Therefore, the inclusion criteria of this study were as follows: (1) subjects who met the diagnostic criteria for MPP; (2) aged 3–6 years old, no gender restrictions; (3) who have normal BMI; (4) who are not receiving any treatment at present; (5) whose parents signed the informed consent form and were willing to cooperate with the investigator.

#### Exclusion criteria

The following are the exclusion criteria of this study: (1) patients who received antibiotics in the last 2 weeks; (2) patients who received probiotics in the last 2 weeks; (3) children with high-fat and high-glucose diet, obesity, or malnutrition; (4) children with other immune disorders and/or on immunomodulatory drugs; (5) pediatric patients with asthma or allergy; (6) pediatric patients with cardiac failure, respiratory failure, hypoxic encephalopathy, and other severe complications; (7) pediatric patients with severe liver, kidney, cardiovascular, endocrine, hematologic, nervous system, and psychiatric disorders; (8) patients who are currently participating in or participated in other clinical trials in the last 3 months; (9) subjects who were prone to lost to follow-up based on the physician’s judgment.

### Sample collection

#### Blood sample collection

A 3 ml of venous blood sample was taken from the subjects on an empty stomach the next morning after being hospitalized into a blood vessel premixed with EDTA and then centrifuged at 4℃ (1000 × g, 10 min). The upper liquid was pipetted out carefully and transferred into a precooled EP tube and was centrifuged at 4℃ (14,000 × g, 15 min). The supernatant was then moved into an enzyme-free EP tube and stored at 80℃ for further analysis.

#### Stool sample collection

1. Fresh stool samples were collected from 130 study subjects, frozen and stored in a −80℃ freezer. The detailed sample collection method and precautions were as follows: (1) Sampling bag instructions: (1) Sampling bag inventory was carried out. The product box was opened and sampling bags were inventoried, including sampling tubes, disposable disinfection cotton swabs, disposable disinfection gloves, instructions for use, and personal information form. (2) Completion of personal information form and informed consent form. (3) Disposable disinfection gloves were removed and worn. (4) The sampling tube was taken out, and the cap was slowly pulled out for sampling. (5) The disposable disinfection cotton swab was used to scoop around 2 g of uncontaminated stools, which were added to the sampling tube. The cap was recapped, and the tube was not opened again. (6) The disposable disinfection cotton swab and disposable disinfection gloves were discarded. The sampling tube, personal information form, and informed consent form were inserted into the sampling bag and submitted to the staff. 2. Precautions: (1) During toileting, the subject sat at the front of the toilet seat as much as possible or hand towels were placed in the toilet bowl to prevent the stool from being soaked in water for a long time, which could affect the accuracy of the results. (2) Results obtained from old stools were considered inaccurate, and stool samples were required to be immediately collected after bowel movements. (3) Transportation requirements: Samples were sealed in self-sealing bags, and the bags were placed in a constant temperature box with ice bags. The temperature of the box was maintained at around −2℃ to prevent gut microbiota destruction due to high sample temperatures.

### DNA extraction, PCR amplification, and high-throughput sequencing

Bacterial DNA was isolated from the stool samples using a DNeasy PowerSoil kit (Qiagen, Hilden, Germany), following the manufacturer’s instructions. DNA concentration and integrity were measured by a NanoDrop 2000 spectrophotometer (Thermo Fisher Scientific, Waltham, MA, USA) and agarose gel electrophoresis, respectively. PCR amplification of the V3-V4 hypervariable regions of the bacterial 16S rRNA gene was carried out in a 25-μl reaction using universal primer pairs (343F: 5′-TACGGRAGGCAGCAG-3′; 798R: 5′-AGGGTATCTAATCCT-3′). The reverse primer contained a sample barcode, and both primers were connected with an Illumina sequencing adapter.

The amplicon quality was visualized using gel electrophoresis. The PCR products were purified with Agencourt AMPure XP beads (Beckman Coulter Co., USA) and quantified using Qubit dsDNA assay kit. The concentrations were then adjusted for sequencing. Sequencing was performed on an Illumina NovaSeq 6000 with two paired-end read cycles of 250 bases each (Illumina Inc., San Diego, CA; OE Biotech Company; Shanghai, China). Paired-end reads were preprocessed using Trimmomatic software^53^ to detect and cut off ambiguous bases (N). It also cuts off low-quality sequences with average quality score below 20 using sliding window trimming approach. After trimming, paired-end reads were assembled using FLASH software^54^. Parameters of assembly were 10 bp of minimal overlapping, 200 bp of maximum overlapping, and 20% of maximum mismatch rate. Sequences were performed further denoizing as follows: reads with ambiguous, homologous sequences or below 200 bp were abandoned. Reads with 75% of bases above Q20 were retained using QIIME software (version 1.8.0)^55^. Then, reads with chimera were detected and removed using VSEARCH^56^. Clean reads were subjected to primer sequences removal and clustering to generate OTU using VSEARCH software with 97% similarity cut off^56^. The representative read of each OTU was selected using QIIME package. All representative reads were annotated and blasted against Silva database (Version 123) using RDP classifier (confidence threshold was 70%)^57^. The 16S rRNA gene amplicon sequencing and analysis were conducted by OE Biotech Co., Ltd. (Shanghai, China).

### Function prediction

A 16S rRNA function prediction was carried out using PICRUSt software for standardization of OTU abundance (i.e., removal of the effects of the 16S marker gene copy number in the species genome). Thereafter, the corresponding Greengenes IDs of each OTU were aligned to the COG and KEGG databases to obtain the COG family information and KEGG Orthology (KO) information corresponding to the OTU. The abundance of each identified COG and KO were calculated. Description and functional information of each COG were obtained from the evolutionary genealogy of genes: Non-supervised Orthologous Groups (EggNOG) based on COG database information to obtain the function abundance spectrum. Information in the KEGG database was used to obtain KO and pathway information, and OTU abundance was used to calculate the abundance of each functional category. The PICRUSt software was used to obtain three levels of metabolic pathway information and abundance at various levels.

### Deoxyribonucleic acid (DNA) copy analysis

Deoxyribonucleic acid (DNA) copy numbers of *Ruminococcus flavefaciens*, *Clostridium butyricum*, *Lactobacillus*, and *Bifidobacterium* in different groups of stool samples were measured with qRT-PCR using the SYBR Green PCR Master Mix (Agilent Technologies, Santa Clara, CA, USA) and then analyzed with the ABI7300 System (Applied Biosystems, Carlsbad, CA, USA). The sequences of primers used were as follows: *Clostridium butyricum*-F (5'-TAAAGGAGTAATCCGCTATG-3') and *Clostridium butyricum*-R (5'-CGTCCCTAAAGACAGAGC-3'), *Ruminococcus flavefaciens*-F (5'-GATGCCGCGTGGAGGAAGAAG-3') and *Ruminococcus flavefaciens*-R (5'-CATTTCACCGCTACACCAGGAA-3'), *Lactobacillus*-F (5'-ACGGGAGGCAGCAGTAGGGA-3') and *Lactobacillus*-R (5'-AGCCGTGACTTTCTGGTTGATT-3'), and *Bifidobacterium*-F (5'-GATTCTGGCTCAGGATGAACGC-3') and *ifidobacterium*-R (5'-CTGATAGGACGCGACCCCAT-3').

### Data analysis

The microbial diversity in stool samples was estimated using the alpha diversity that includes Chao1 index^58^ and Shannon index^59^. Principal coordinate analysis (PCoA) was computed based on Bray-Curtis or Binary-Jaccard distance matrices and permutational multivariate analysis of variance (Adonis test) were used to compare the overall dissimilarity of microbiota among different groups of stool samples, drawing with online tools (https://cloud.oebiotech.com/task/).

Statistical analysis for DNA copy number was performed using GraphPad Prism 8.4.2 (GraphPad Software, USA) and expressed as mean ± standard deviation. Whether the data were normally distributed was tested using the Shapiro-Wilk test. One-way analysis of variance (ANOVA) test was performed among three groups. If the data were not normally distributed, comparisons were performed by Kruskal-Wallis test. *P* < 0.05 was considered statistically significant.

### Ethics approval and consent to participate

All study tasks were carried out in accordance with the Declaration of Helsinki (1964). The study was approved by the Ethics Committee of Longhua Hospital Affiliated to Shanghai University of Traditional Chinese Medicine (No: 2019LCSY018). All methods were performed in accordance with the relevant guidelines and regulations. All study participants provided written consents for future research, and guardians provided the consents on behalf of patients under 8 years old.

## Supplementary Information

Supplementary Information.
